# *Yarrowia lipolytica* Extracellular Lipase Lip2 as Biocatalyst for the Ring-Opening Polymerization of ε-Caprolactone

**DOI:** 10.3390/molecules22111917

**Published:** 2017-11-07

**Authors:** Karla A. Barrera-Rivera, Antonio Martínez-Richa

**Affiliations:** Departamento de Química, División de Ciencias Naturales y Exactas, Universidad de Guanajuato, Noria Alta S/N, Colonia Noria Alta, Guanajuato, Guanajuato 36050, Mexico

**Keywords:** *Yarrowia lipolytica*, lipases, lipase immobilization, enzymatic polymerization

## Abstract

*Yarrowia lipolytica* (YL) is a “non-conventional” yeast that is capable of producing important metabolites. One of the most important products that is secreted by this microorganism is lipase, a ubiquitous enzyme that has considerable industrial potential and can be used as a biocatalyst in the pharmaceutical, food, and environmental industries. In this work, *Yarrowia lipolytica* lipase (YLL) was immobilized on Lewatit and Amberlite beads and is used in the enzymatic ring-opening polymerization (ROP) of cyclic esters in the presence of different organic solvents. YLL immobilized on Amberlite XAD7HP had the higher protein adsorption (96%) and a lipolytic activity of 35 U/g. Lewatit VPOC K2629 has the higher lipolytic activity (805 U/g) and 92% of protein adsorption. The highest molecular weight (Mn 10,685 Da) was achieved at 90 °C using YLL that was immobilized on Lewatit 1026 with decane as solvent after 60 h and 100% of monomer conversion.

## 1. Introduction

The non-conventional yeast *Yarrowia lipolytica* is of interest for fundamental research and biotechnological applications. The fundamental studies play a crucial role in the establishment and development of the biotechnological processes. *Yarrowia lipolytica* is widespread in nature. Since *Y. lipolytica* is lipophilic and oleophilic yeast, the yeast strains are easily isolated from different sources containing lipid and hydrocarbon compounds, such as oily food and natural environments like oil fields [1]. The maximum growth of most *Yarrowia* strains is below 32–34 °C, and the yeast is not considered to be a possible human pathogen. *Y. lipolytica* has been classified as Generally Regarded. As Safe (GRAS) by the American Food and Drug Administration (FDA) [2]. *Yarrowia lipolytica* is a good model organism for protein secretion studies. *Y. lipolytica* secretes a set of valuable proteins, such as alkaline or acid proteases, RNases, phosphatases, lipases and inulinase into the medium, which are interesting for biotechnological applications. The enzymes could be used in the detergent, food, pharmaceutical, and environmental industries. The protein secretion pathway is also important to heterologous protein secretion by recombinant strains of *Y. lipolytica* [3].

Lipases (E.C. 3.1.1.3) are serine hydrolases defined as triacylglycerol acylhydrolases. They catalyze the hydrolysis of the ester bond of tri-, di-, and monoglycerides of long-chain fatty acids into fatty acids and glycerol. They differ from esterase (EC 3.1.1.1) due to their ability to hydrolyze triglyceride at the lipid-water interface [4].

Lipases are primarily responsible for the hydrolysis of acylglycerides. However, a number of other low- and high-molecular weight esters, thiol esters, amides, and polyol/polyacid esters are accepted as substrates by this unique group of enzymes [5].

Lipases secretion in *Y. lipolytica* was first reported in 1948 by Peters and Nelson [6,7], who described a single glucose-repressible activity with a pH optimum of around pH 6.2–6.5. Ota et al. described both an extracellular lipase activity in cultures supplemented with a protein-like fraction derived from soybean, and two cell-bound lipases: lipase I (39 kDa) and lipase II (44 kDa). The extracellular lipase required oleic acid as stabilizer-activator, whereas the cell-bound lipases did not and differed by several properties from the extracellular enzyme [8,9].

In 1993, it was first demonstrated that medium size lactones, δ-valerolactone (δ-VL, 6-membered), and ε-caprolactone (ε-CL, 7-membered), were polymerized by industrial lipases derived from *Candida cylindracea* (lipase CC), *Burkholderia cepacia* (lipase BC), *Pseudomonas fluorescens* (lipase PF), and porcine pancreas (PPL) [10,11,12]. *Candida antartica* (lipase CA), *Candida rugosa* (lipase CR), and *Rhizomucor meihei* (lipase RM) were also active for ROP of these monomer [13]. Ring-opening polymerization of various unsubstituted and substituted lactones, as well as other cyclic monomers has been extensively studied [14,15,16,17,18,19,20,21,22,23].

### Yarrowia lipolytica Lipases as Biocatalysts

Lipases have emerged as one of the leading biocatalysts with proven potential for contributing to the multibillion-dollar lipid technology bio-industry. *Yarrowia lipolytica* has been considered as an industrial workhorse because of its ability to produce important metabolites and intense secretory activity. One of the most important products secreted by this microorganism is lipase.

Our laboratory previously isolated a stable lipase from this yeast. The effect of used commercial oil from a vacuum pump (instead of olive oil) and the presence of wheat flour were evaluated [24].

In this work, the ROP of ε-caprolactone by immobilized lipase from *Y. lipolytica* in the presence of organic solvents was investigated for the first time. The effects of lipase concentration (6–72 mg), monomer concentration (0.6–6 mmol), and temperature (70, 90 and 120 °C) were evaluated.

## 2. Results and Discussion

### 2.1. Lipase Isolation and Immobilization

Lewatit VPOC K3433 had the lower protein adsorption (18%) and the lower lipolytic activity (3 U/g). Amberlite XAD7HP had the higher protein adsorption (96%) and a lipolytic activity of 35 U/g. Lewatit VPOC K2629 has the higher lipolytic activity (805 U/g) and 92% of protein adsorption.

For styrene resin beads the saturation time for YLL absorption was ~60 min. The adsorption rates of styrenic resins are attributed to stronger hydrophobic interactions between styrenic surfaces, functional groups of the resins and YLL. The dependence of adsorption rate on particle size is due to the pore size that is limiting protein transport to the inside of the particles. The small size of pores slows protein diffusion into beads so that smaller beads more rapidly were saturated in protein. Results for protein immobilization are summarized in Table 1.

### 2.2. Enzymatic Synthesis of Poly (ε-Caprolactone) Using Immobilized Y. lipolytica Lipase

As we reported previously, free *Y. lipolytica* lipases are efficient catalysts for the polymerization of lactones in the presence of n-heptane, but the reaction times are long [10].

**Figure 1:**
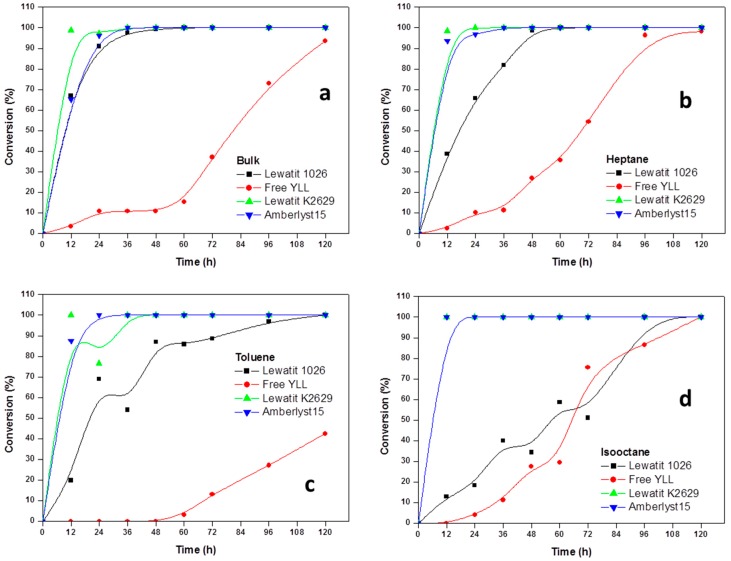

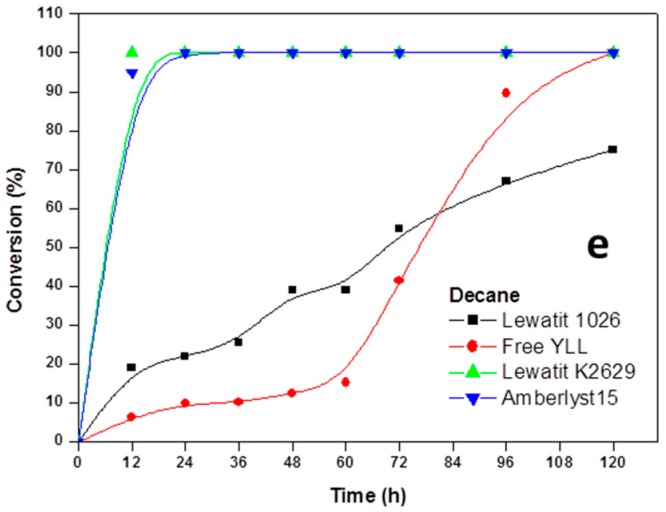
Effect of reaction time on monomer conversion. Polymerization in the presence of organic solvents. R = 1.08 mmol ε-CL/12 mg of immobilized lipase at 70 °C. Plots with the highest monomer conversions from lipase immobilized on Lewatit and Amberlite beads. (**a**) Polymerization in bulk; (**b**) Polymerization in the presence of heptane; (**c**) Polymerization in the presence of toluene; (**d**) Polymerization in isooctane; and, (**e**) Polymerization in decane.

Experiments were performed to determine the reaction order of monomer. The effect of incubation times in the polymerization of ε-CL at 70 °C was done and the results are shown in Figure 1. It was found that lipases that are immobilized on amberlite beads are not efficient in the enzymatic polymerization of ε-CL. We can observe that 100% of monomer conversion was reached at 12 h when *Y. lipolytica* lipase was immobilized in Lewatit K2629 (using decane as solvent, Mn 778 Da; isooctane, Mn 892 Da; Toluene, Mn 673 Da) and amberlyst 15 (isooctane, Mn 930 Da). Lowest conversion (0%) was seen when free *Y. lipolytica* lipase was used in toluene and isooctane. The highest molecular weights were obtained when *Y. lipolytica* lipase was immobilized in Lewatit 1026 and toluene was used as solvent at 60 h of reaction (Mn 7441 Da, 86% monomer conversion); the lowest molecular weight was reached at 120 h of reaction using free *Y. lipolytica* lipase without solvent (Mn 683 Da, 42% of monomer conversion).

The time dependence of the polyesterification in four different solvents, showed the general trend of increasing the degree of polymerization with time.

Based on the kinetic curves, we conclude that immobilized lipases from *Y. lipolytica* are efficient catalysts for the polymerization of lactones in the presence of organic solvents.

**Figure 2:**
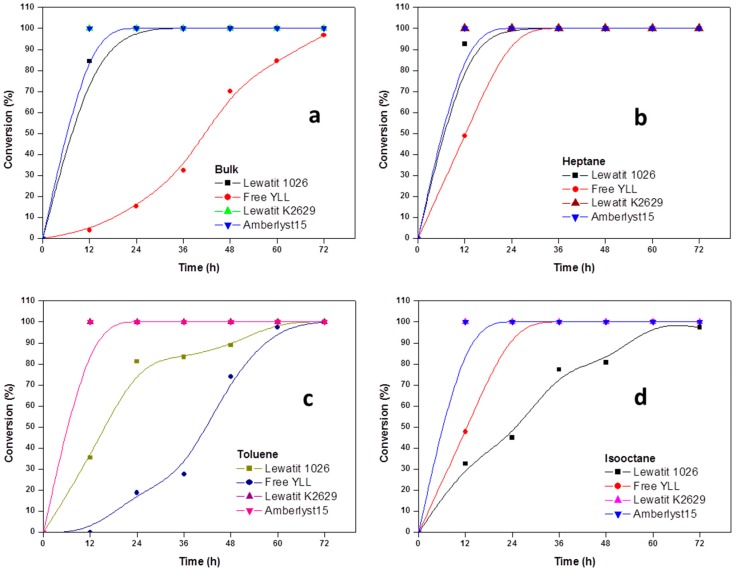

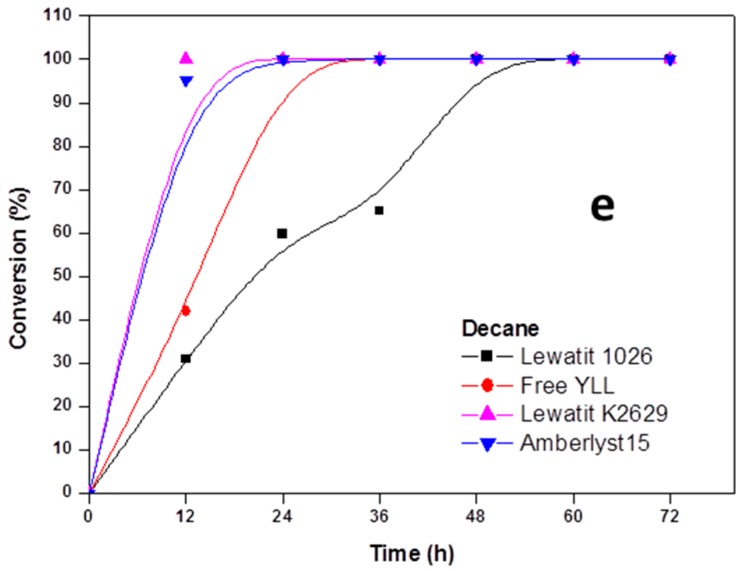
Effect of reaction time on monomer conversion. (**a**) Polymerization in bulk; (**b**) Polymerization in the presence of heptane; (**c**) Polymerization in the presence of toluene; and, (**d**) Polymerization in isooctane; (**e**) Polymerization in decane. R = 1.08 mmol ε-CL/12 mg of immobilized lipase at 90 °C.

In Figure 2, the results for enzymatic polymerizations at 90 °C are shown. We can observe that 100% of monomer conversion was reached after 12 h of reaction time when *Y. lipolytica* lipase was immobilized on Lewatit K2629 beads (bulk, Mn 899 Da; heptane, Mn 974 Da; toluene, Mn 863 Da; isooctane, Mn 1493 Da; decane, Mn 1441 Da); when free YLL was used, a conversion of 100% was reached at 72 h, and a Mn 1095 in bulk conditions. The highest molecular weight (Mn 10,685 Da) was obtained after 60 h (100% monomer conversion) using YLL that was immobilized on Lewatit 1026 and using decane as solvent, being the lowest molecular weight (Mn 708 Da) using free YLL in toluene after 48 h and a monomer conversion of 74%.

When YLL was immobilized on amberlyst 15, 100% of monomer conversions were reached after 12 h (bulk, heptane, toluene, isooctane, and decane). The highest molecular weight was obtained after 72 h in heptane (Mn 3369 Da) and the lowest after 12 h in toluene (Mn 862 Da).

The dependence of the catalytic activity of the enzymes on the polarity of solvents is attributed to the solvent’s ability to strip the enzyme from its essential water layer, or to penetrate into the water layer in order to distort the interaction between the water and the enzyme molecule. Polyesters of higher molecular weight were obtained in poor solvents, whereas in bulk and in good solvents only low-molecular weight oligomers were formed. This latter finding could be explained in terms of the good solubility and the consequent removal of the product oligomers from the enzyme surface, resulting in a lowering of the concentration of the oligomer-substrate near the enzyme. However, in a poor solvent, the growing substrate concentrates near the enzyme, and thus enables further reaction between the functional hydroxyl and ester groups of larger molecules, and consequently higher-molecular-weight polyesters were produced.

By raising the reaction temperature, a sharp increase in the degree of polymerization of the product polyesters could be observed. A sharp increase in the monomer conversion could be observed too. When comparing experiments carried out at prolonged times at 70 and 120 °C, showed that the molecular weights of the obtained polymers were about 683 and 3498 Da, respectively.

**Figure 3:**
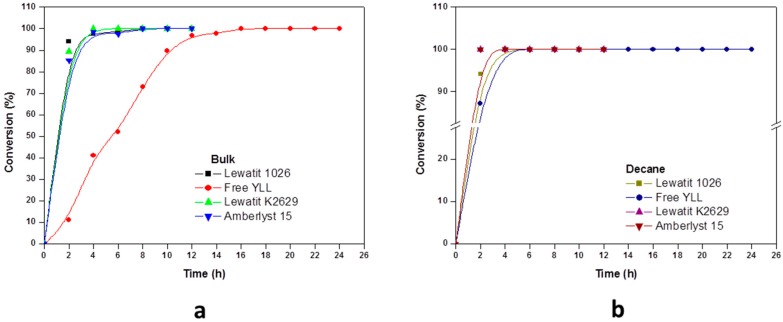
Effect of reaction time on monomer conversion. (**a**) Polymerization in bulk and (**b**) Polymerization in the presence of decane. R = 1.08 mmol ε-CL/12 mg of immobilized lipase at 120 °C.

In Figure 3, the results for enzymatic polymerizations at 120 °C are shown. A control was performed where no enzyme was used. In this control, no monomer conversion was observed after 24 h, and no polymer could be isolated. Monomer conversion of 100% was reached at 2 h using YLL immobilized on Lewatit K2629 (Mn 736 Da), and amberlyst 15 (Mn 788), and decane as solvent. When bulk conditions were used, free YLL reached the 100% of monomer conversion at 16 h and a molecular weight of 1155. The highest molecular weight (Mn 3498 Da) was obtained after 8 h using bulk conditions and YLL immobilized on Lewatit 1026 (100% monomer conversion); the lowest molecular weight (Mn 1059 Da) was obtained using free YLL after 24 h and using decane as solvent (100% monomer conversion). Lip2 is expressed as a 301 amino acid glycosylated protein of 38 kDa [25], this glycosylation protects the enzyme from denaturation at higher reaction temperatures.

### 2.3. Enzyme Concentration

**Figure 4:**
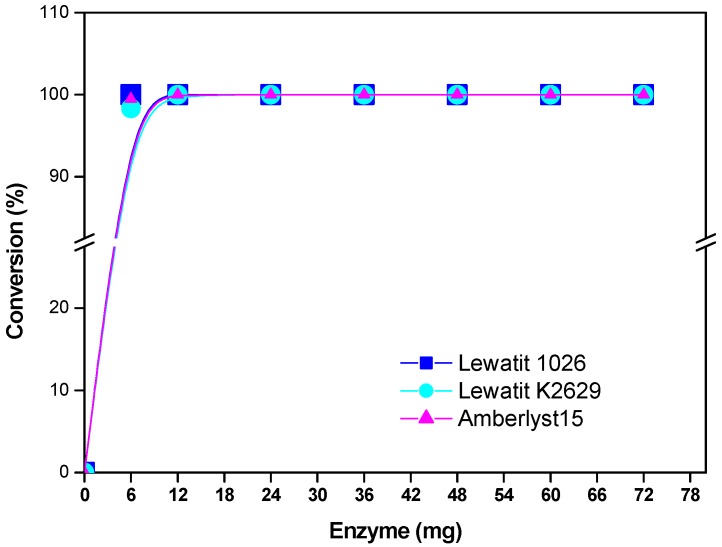
Effect of enzyme concentration on monomer conversion. Polymerization in the presence of decane. R = 1.08 mmol ε-CL/x mg of immobilized lipase (x = 6, 12, 24, 36, 48, 60, and 72 mg) at 120 °C and 12 h.

Figure 4 shows that by increasing the catalyst concentration, the percent of monomer conversion rate increased. From a series of kinetic experiments, it was determined, that, by increasing enzyme/substrate (E/S) concentration, higher molecular weights were attained in a relatively shorter reaction time. Since the enzyme component of the reaction mixture normally has the relatively highest molecular weight percent of water, it follows that higher E/S ratios will lead to greater reaction water contents, which acts as a chain-transfer agent with the growing chain.

### 2.4. The Effect of the Substrate Concentration

**Figure 5:**
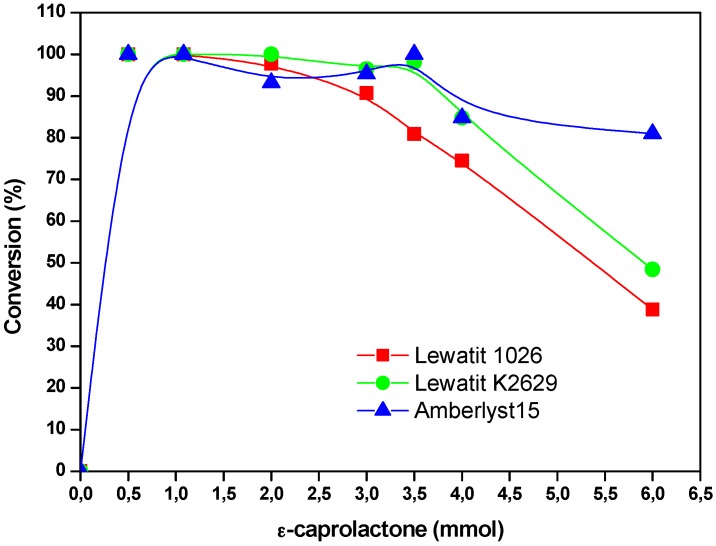
Effect of the concentration of the substrate on monomer conversion. Polymerization in the presence of decane. R = x mmol ε-CL/12 mg of immobilized lipase at 120 °C and 10 h.

The concentration of the cyclic ester in the solvent (decane), influences the degree of polymerization of the obtained polymer but also the formation of macrolactones. At lower concentrations, monomer conversion of 100% is observed. The highest molecular weight was obtained when YLL was immobilized on Lewatit 1026 (Mn 4722 Da) and 2 mmol of the substrate was used. When YLL was immobilized on amberlyst 15, a Mn of 2014 was observed when 6 mmol of the substrate was used. It was found that monomer conversion decreases when substrate concentration was higher (Figure 5).

## 3. Materials and Methods

ε-caprolactone (ε-CL), was dried over calcium hydride and distilled under reduced pressure before use. Heptane 99%, toluene 99%, isooctane 99%, decane 99%, Lewatit VP OC 1026, Lewatit VP OC K2629, Lewatit CNP-105, Lewatit VP OC 1064 MD PH, Lewatit VP OC 1163, Lewatit VP OC 1065 weakly basic, Lewatit MP 62 free base, Lewatit monoplus TP 214, Lewatit VP OC K3433, Lewatit SP 112, Amberlyst 15, Amberlite XAD 16, Amberlite XAD 7HP, Amberlite XAD 1180, Amberlite XAD4, ethanol, distilled water, Chloroform-*d* (CDCl_3_), olive oil, malt extract, CaCO_3_, MgSO_4_, K_2_HPO_4_, urea, soybean meal, glucose, wheat flour, (NH_4_)_2_SO_4_, and corn steep liquor (CSL) were purchased from Sigma-Aldrich and used as received.

### 3.1. Organism

*Y. lipolytica* strain was used in this study. It was maintained on slants of solid YPG medium (0.3% yeast extract, 1% peptone, 2% glucose, and 2% agar, pH 6.3), as previously described by Bartnicki-García and Nickerson [26].

### 3.2. Protein Determination

Protein was measured by the method of Bradford [27] using bovine serum albumin as standard. The Bradford assay is a protein determination method that involves the binding of Coomassie Brilliant Blue G-250 dye to proteins. The dye exists in three forms: cationic (red), neutral (green), and anionic (blue) (Compton and Jones 1985). Under acidic conditions, the dye is predominantly in the doubly protonated red cationic form (A_max_ = 470 nm). However, when the dye binds to protein, it is converted to a stable unprotonated blue form (A_max_ = 595 nm). It is this blue protein-dye form that is detected at 595 nm in the assay using a spectrophotometer or microplate reader.

### 3.3. Lipase Activity

Lipase activity was measured using the simplified method of *para*-nitrophenyl palmitate assay for lipases and esterases, as previously described by Gupta et al. [28]. The basis of this assay protocol is the colorimetric estimation of *para*-nitrophenol (*p*NP) released as a result of enzymatic hydrolysis of *p*NPP at 410 nm. One unit of enzyme activity is defined as the amount of enzyme liberating 1 μmol of *p*-nitrophenol per minute.

### 3.4. Lipase Isolation and Immobilization

Lipase production by *Yarrowia lipolytica* was made, as previously reported by Barrera et al., SDS-PAGE 12% showed one main protein band of 44.7 kDa revealed by silver stain [24]. Before immobilization, Lewatit, amberlite, and amberlyst beads were activated with ethanol (1:10 beads: ethanol) for 5 h, washed with distilled water and dried under vacuum for 24 h at room temperature. The beads (1 g) were shaken in a rotatory shaker in 15 mL of YLL lipase solution (the broth obtained after incubation and centrifugation to remove the yeast cells) with 0.16 mg/mL and pH 7.0 at 4 °C for 14 h. After incubation, the beads were filtered off, washed with distilled water, and then dried under vacuum for 24 h at room temperature.

### 3.5. Synthesis of Poly (ε-Caprolactone) Using Immobilized Y. lipolytica Lipase

Polymerizations were carried out by duplicate in 10-mL stoppered vials previously dried and purged with dry nitrogen. In a typical run, monomer (ε-CL, 1.08 mmol), catalyst (enzyme, 12 mg) and solvent (1 mL) were added under dry nitrogen atmosphere. Polymerizations were also carried out in bulk conditions. Vials were stoppered with a rubber septum and placed in a thermostated bath at predetermined temperatures (70, 90 and 120 °C) and time periods (12, 24, 36, 48, 54, 60, 72 and 120 h). Molecular weights and conversions during reaction were monitored by ^1^H-NMR. Solution ^1^H-NMR spectra were recorded at room temperature on a Varian Gemini 200 (200 MHz). Chloroform-*d* (CDCl_3_) was used as solvent. Spectra were referenced to the residual solvent protons at δ = 7.26 ppm for CDCl_3_ in the ^1^H-NMR spectrum. Degree of polymerization and monomer conversion were determined by ^1^H-NMR from the relative peak areas of signals corresponding to the ester methylene of the polymer (t, δ = 4.04 ppm CH_2_OCO), the ester methylene of the monomer (t, δ = 2.6 ppm CH_2_COO), and the chain terminal methylene groups (t, δ = 3.6 ppm CH_2_OH).

## 4. Conclusions

*Yarrowia lipolytica* lipase (YLL) (free and immobilized in Lewatit and Amberlite resins) has been tested as a biocatalyst for the ring-opening polymerization of cyclic esters in different organic solvents. Effect of monomer concentration, lipase concentration, solvent, and temperature were evaluated. Reactions carried out in poor solvents (such as hexane and decane) produce higher yields and molecular weights. On the other hand, good solvents deliver lower outputs. Higher temperatures increase the rate of polymerizations and molecular weights. Maximum monomer conversions are achieved when 0.5–3 mmol are used in the presence of 12 mg de immobilized lipase al 120 °C.

## 5. Future Remarks

In this work, the application of *Yarrowia lipolytica* lipase biocatalysis to the production of biodegradable polyesters with a wide range of molecular weights has been explored. Following this approach, synthesis of other polymer derivatives (such as biodegradable polyurethanes and polyureas) is currently being explored in our research group. *Yarrowia lipolytica* lipase has found widespread biotechnology applications in the food, pharmaceutical, detergent, and other industries [29]. As a result, applications of lipase biocatalysis to produce different chemical materials have increased in recent years and are expected to continue in the future. As this process is based in a clean biocatalytic technology, in a long-term perspective it can be used to develop environmentally benign processes at the industrial level with minimal waste generation. Our approach provides of alternative chemical routes to obtain biodegradable polymers in an early stage of development, on the basis of a green chemistry approach. The used methodology can render products with improved properties produced in milder conditions. An important disadvantage of lipase biocatalysis for industrial applications is their low long-term stability under reaction conditions and the difficult recovery and recycling. However, if the enzyme is immobilized on a suitable support, such as the resins used in this work, a series of stable biocatalytic systems can be obtained. This approach is also tunable to variations in the synthesis conditions. Hence, the next level of research for this biocatalytic system is to design processes, which lend themselves to facile recovery and recycling of the catalyst, with good product yields and improved properties (as compared to those observed for polymers obtained by traditional chemical process).

## Figures and Tables

**Table 1:** Matrix parameters and loading of *Yarrowia lipolytica* lipase on Lewatit, Amberlite and Amberlyst beads.

Resin	Protein Content (mg/g)	Protein Adsorption (%)	Protein Activity (U/g) *
Lewatit VPOC 1026	0.14	87	47
Lewatit VPOC K2629	0.14	92	805
Lewatit CNP-105	0.14	88	291
Lewatit 1064 MD PH	0.10	42	137
Lewatit VPOC 1163	0.10	52	86
Lewatit VPOC 1065 weakly basic	0.11	69	19
Lewatit MP62 free base	0.10	50	81
Lewatit monoplus TP214	0.14	88	23
Lewatit VPOC K3433	0.02	18	3
Lewatit SP112	0.10	51	144
Amberlite XAD16	0.11	69	31
Amberlite XAD7HP	0.15	96	35
Amberlite XAD1180	0.15	95	5
Amberlite XAD4	0.10	64	72
Amberlyst 15	0.12	74	512

* One unit of lipase activity was defined as the production of 1 µmol of *ρ*-nitrophenol per minute.
